# Langerhans cell histiocytosis mimicking hematoma of the lower eyelid: A case report

**DOI:** 10.1097/MD.0000000000041039

**Published:** 2024-12-20

**Authors:** Jing Li, Mingyu Ren, Jianjie Wang, Yanyan Cheng, Ruimiao Li, Lu Lu, Ruina Zhang

**Affiliations:** a Intensive Care Unit, Xingtai Central Hospital, Xingtai, Hebei, China; b Department of Orbital Disease and Ocular Tumor, Hebei Eye Hospital, Xingtai, Hebei, China.

**Keywords:** case report, hematoma, langerhans cell histiocytosis, lower eyelid

## Abstract

**Rationale:**

The orbital Langerhans cell histiocytosis (LCH) is rare clinically, due to its ability to mimic other conditions, distinguishing LCH from hematoma when these disorders coexist can be particularly challenging.

**Patient concerns:**

A 3-year-old boy presented with a 2-week history of unresolved bruising and swelling of the left eye. CT revealed a well-defined cystic lesion in the preseptal tissues of the left eyelid, with an incomplete bone structure at the lower margin of the orbit. MRI revealed a well-circumscribed circular mass in the lower margin of the orbit, and a second lesion under the hematoma.

**Diagnosis:**

Postoperative histological examination revealed a diagnosis of LCH with concurrent hematoma.

**Interventions:**

The lesions in the left eye were surgically removed through a lower eyelid skin incision under general anesthesia. The patient received 6 courses of systemic chemotherapy, consisting of a combination of vinblastine and prednisolone.

**Outcomes:**

No recurrence was observed after a follow-up period of 11 months.

**Lessons:**

LCH rarely occurs at the infraorbital margin. When external factors, lead to local bleeding and hematoma formation, the presence of lesions may be masked, resulting in a missed diagnosis. Radiographic features such as localized “worm-eaten” bone destruction should not be overlooked for timely LCH diagnosis and treatment.

## 1. Introduction

Langerhans cell histiocytosis (LCH) is an inflammatory neoplasia of myeloid precursor cells driven by mutations in the mitogen-activated protein kinase pathway.^[1]^ While LCH can involve any organ individually or concurrently, the bones and the skin are the most affected. However, orbital LCH is particularly rare clinically. Of note, LCH can exhibit substantial variability in its clinical presentation, often mimicking different conditions.^[2]^ Due to its rarity and some clinicians’ lack of a comprehensive understanding of its clinical and imaging characteristics, especially when it is mixed with other changes, orbital LCH poses diagnostic challenges.

## 2. Case report

A 3-year-old boy presented with a 2-week history of unresolved bruising and swelling of his left eye. He had previously fallen and injured the left side of his face when he accidentally struck the corner of a table. Despite 2 weeks of treatment for eyelid hematoma, local swelling did not subside. Consequently, the patient was referred to our department for further treatment.

**Figure 1.**
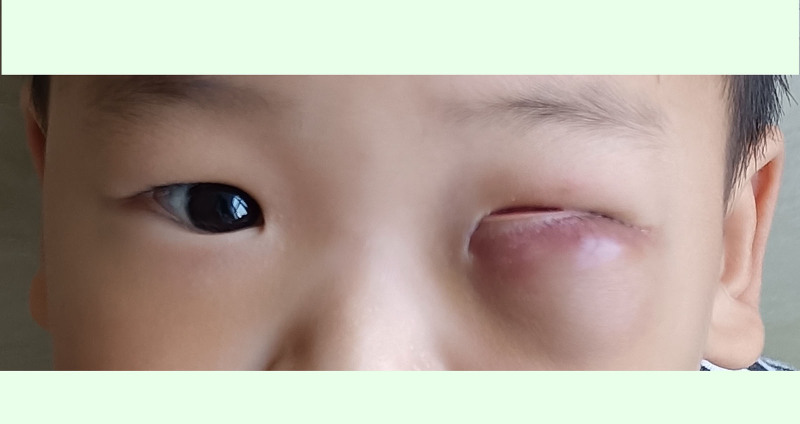
The left lower eyelid is highly swollen.

Given the patient’s age, physical examination was quite challenging. The left lower eyelid is highly swollen, with a highly elevated lesion obstructing the eyeball and hindering visualization of other ocular structures (Fig. 1). The examination of the right eye did not reveal any abnormalities. In addition, a fundus examination of the right eye showed no abnormalities, and no neurological signs were observed during the physical examination. No abnormalities were found in the physical examination of other parts of the body. There were no known underlying medical conditions or relevant family medical history.

**Figure 2.**
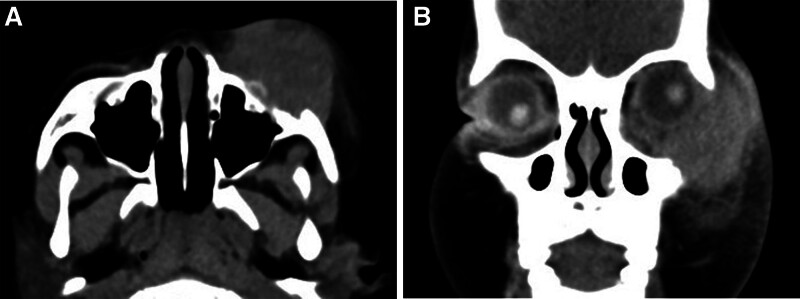
Orbital CT showed a well-defined cystic lesion in the preseptal tissues of the left eyelid, with an incomplete bone structure at the lower margin of the orbit. (A) Axial CT scan; (B) Coronal CT scan. CT = computed tomography.

**Figure 3.**
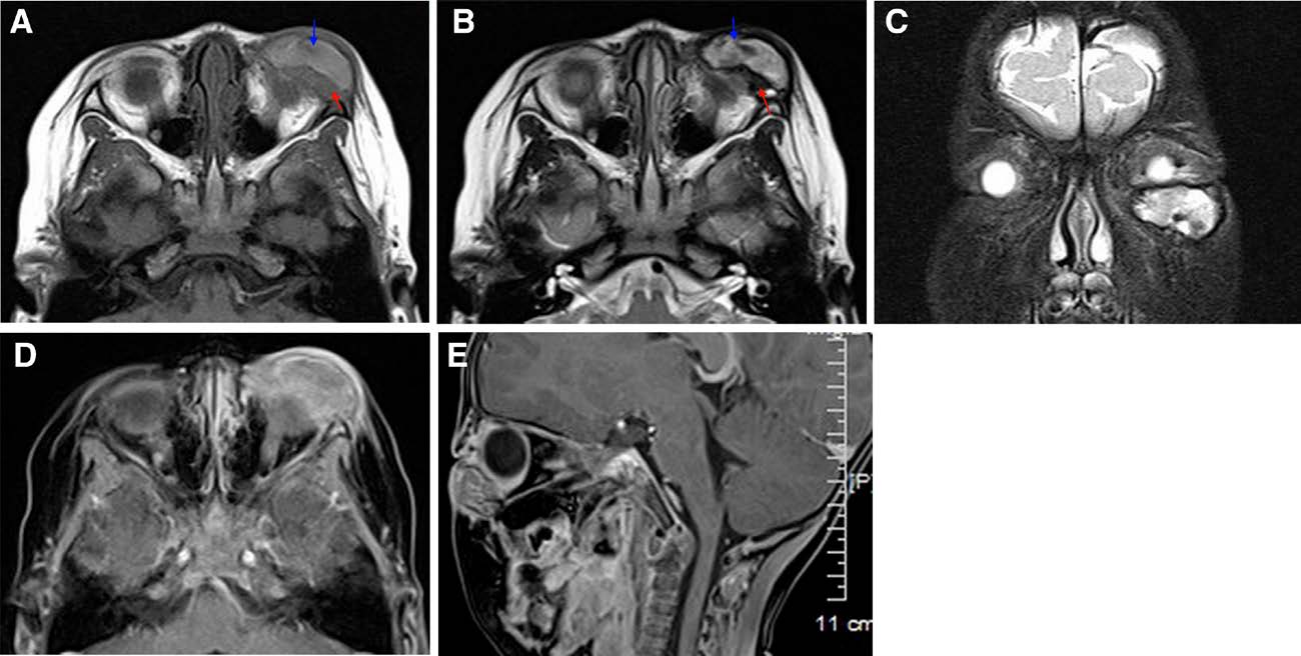
Orbital MRI revealed a well-circumscribed circular mass in the lower margin of the orbit (Blue arrow), and a second lesion under the hematoma and adjacent to the damaged bone (Red arrow). (A) Axial MRI scan on T_1_WI; (B) Axial MRI scan on T_2_WI; (C) Coronal MRI scan on fat-suppressed T_2_WI; (D) Axial MRI scan on contrast-enhanced T_1_WI; (E) Sagittal MRI scan on contrast-enhanced T_1_WI. MRI = magnetic resonance imaging, T1WI = T1 weighted images, T2WI = T2 weighted images.

Orbital computed tomography (CT) imaging performed on the day of the patient’s injury revealed a well-defined cystic lesion in the preseptal tissues of the left eyelid, with an incomplete bone structure at the lower margin of the orbit. The mass measured approximately 4.12 cm × 2.88 cm × 2.07 cm (Fig. 2). Orbital magnetic resonance images (MRI) after admission, revealed a well-circumscribed circular mass in the lower margin of the orbit. On T_1_ weighted images (T_1_WI), the lesion exhibited hyperintensity or mixed signals, while on T_2_ weighted images (T_2_WI), it primarily displayed isointense and hyperintense signals. Contrast-enhanced imaging revealed that the mass was most likely to be a hematoma since it was not enhanced. Meanwhile, a second lesion under the hematoma and adjacent to the damaged bone showed an isointense signal on T_1_WI and hypointense mixed signals on T_2_WI. Contrast-enhanced imaging showed that part of this lesion was slightly enhanced (Fig. 3). The imaging examinations suggested that there may be neoplastic lesions or tuberculous osteomyelitis under the hematoma. The provisional diagnosis was the orbital masses with hematoma.

To rule out the possibility of neoplastic lesions, and the occurrence of amblyopia caused by long-term lesions covering the eyeball, the lesions in the left eye were surgically removed through a lower eyelid skin incision under general anesthesia. During the operation, the large hematoma was first excised, revealing a dark-purple and irregularly shaped lesion coupled with osteolytic changes in the bone of the inferior orbital margin. Notably, the orbital septum and periosteum remained intact throughout the procedure. Due to the limited lesions and damaged bone, we successfully excised the lesions and employed a micro-dynamic system to remove the affected bone. Finally, the surgical area was thoroughly irrigated with normal saline.

**Figure 4.**
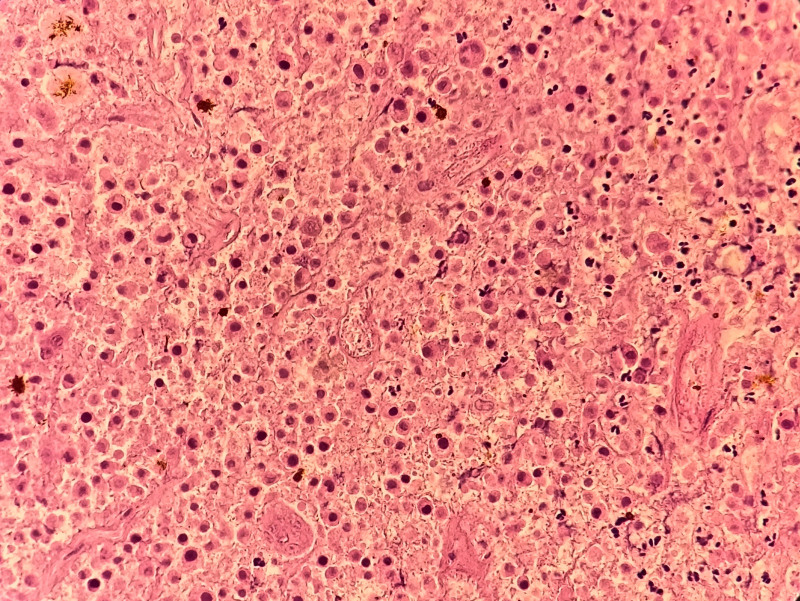
Histological examination revealed a diagnosis of LCH (hematoxylin and eosin staining, 100×). LCH = langerhans cell histiocytosis.

**Figure 5.**
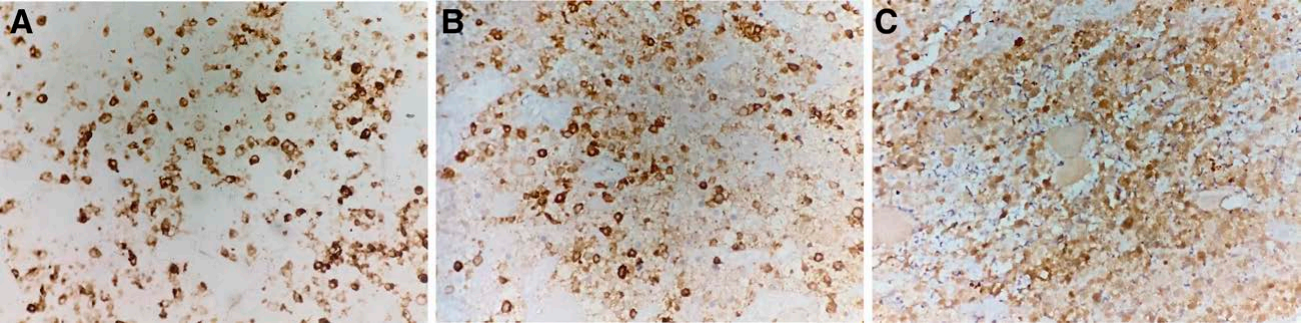
Immunohistochemical examinations of lesions. (A) Tumor cells positive for Langerin (400×); (B) Tumor cells positive for CD1a (400×); (C) Tumor cells positive for S-100 (400×).

Histological examination revealed a diagnosis of LCH in concurrence with hematoma (Fig. 4). Immunohistochemical staining was positive for Langerin, CD1a, S-100, CD68, CD4, CD31, CD45, CD163, CD2, P53 (1%), and Ki-67 (5%), while being negative for CD15, CD30, Desmin, Melan-A, and CD56 (Fig. 5).

**Figure 6.**
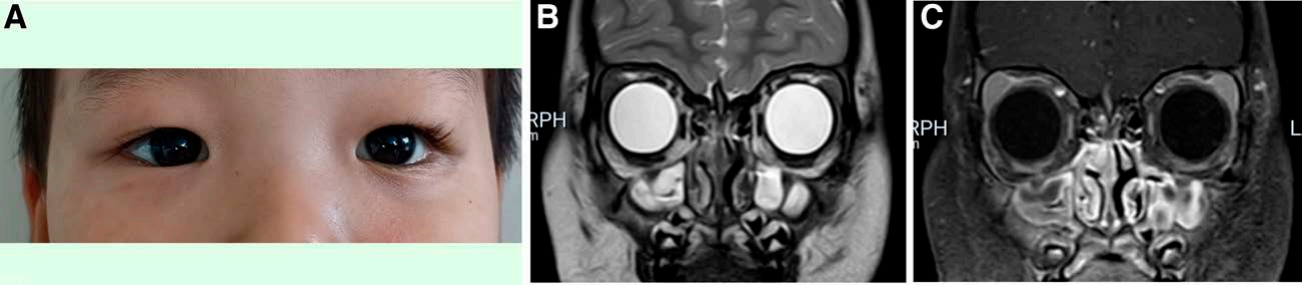
Results of patient review. (A) The appearance of the patient is normal; (B) No tumor recurrence was found on coronal MRI scan on T_2_WI; (C) No tumor recurrence was found on contrast-enhanced T_1_WI. MRI = magnetic resonance imaging, T_1_WI = T_1_ weighted images, T_2_WI = T_2_ weighted images.

The patient was subsequently referred to the Department of Pediatric Oncology at another medical institution to further rule out any systemic lesions. Positron-emission tomography (PET) imaging was performed, revealing no other lesions. Because pediatricians believe that orbital invasion is a high-risk factor for the disease, following this, the patient received a total of 6 courses of systemic chemotherapy comprising a combination of vinblastine and prednisolone therapy in the Department of Pediatric Oncology. During the 11-month follow-up period, no evidence of recurrence was reported (Fig. 6).

## 3. Discussion

LCH is a clinically heterogeneous condition, ranging from self-resolving skin or single bone lesions to systemic forms involving the bone marrow, liver, and/or spleen.^[3]^ Isolated orbital lesions are occasionally encountered in clinical practice. It can occur at any age but is more prevalent in children between 5 and 10 years old.^[4]^ Orbital LCH lesions typically involve the superior or superolateral orbital roof and are superficially located.^[5]^ Imaging examinations often reveal a soft tissue mass and bony defects as well as orbital tumors and intracranial involvement.^[4]^ While identifying LCH can be difficult for many clinicians, diagnosing orbital LCH becomes less challenging if 1 is familiar with its clinical features and imaging findings. However, patients with a history of trauma may easily be misdiagnosed as having a hematoma, complicating the diagnosis. Herein, the lesion appeared at the zygomaticomaxillary suture of the inferior orbital margin rather than the more common locations of the superior or superolateral orbital roof. Moreover, the relatively small lesion was concealed by a superficial hematoma. The lesion beneath the hematoma exhibited an isointense signal on T_1_WI and hypointense mixed signals on T_2_WI, with contrast-enhanced imaging revealing slight enhancement in part of the lesion. Worm-eaten bony changes were present at the inferior orbital region, which is significantly different from fractures. Together, these findings align with the characteristic imaging features of LCH and could help physicians distinguish it from hematomas. Furthermore, in our opinion, the occurrence of a hematoma at this site may be associated with LCH, due to LCH makes the local tissues more susceptible to external forces.

Moreover, there is a need to differentiate LCH from orbital cholesterol granuloma. Cholesterol granuloma is characterized by an osteolytic lesion with a granulomatous reaction, including cholesterol crystals, and is commonly surrounded by a fibrous capsule.^[6]^ Orbital cholesterol granuloma generally occurs in the frontal bone in the superolateral aspect of the orbit.^[7]^ On CT scans, cholesterol granuloma appears as a cystic lesion with irregular bone destruction, making it quite similar in appearance to LCH. However, regarding MRI findings, cholesterol granulomas typically exhibit hyperintense signals on both T_1_WI and T_2_WI. Besides, contrast-enhanced imaging does not usually reveal significant enhancement. Additionally, the site of onset can be used as a basis for distinguishing between cholesterol granuloma and LCH.

Treatment perspectives in LCH are influenced by dissimilar patient encounters and varied interpretations of the disease process.^[8]^ The current classification differentiates between single-system disease (SS-LCH) and multisystem disease (MS-LCH), a distinction based on the extent of involvement at diagnosis.^[9]^ Most physicians commonly accept the perspective that patients with single lesions often exhibit a favorable response to localized treatments, while those with multisystem disease and organ involvement typically necessitate more intensive therapy.^[10]^ Thus, local therapies are recommended for SS-LCH with isolated skin or bone involvement, with surgical excision being the preferred treatment of choice. In the case of MS-LCH or SS-LCH with multifocal bone lesions, the consensus strongly favors systemic therapy. While no universally accepted standard treatments exist, a combination of vinblastine and prednisolone therapy is a commonly used regimen, particularly in pediatric LCH cases.^[11]^ Orbital lesions with intracranial extension are classified as CNS lesions, necessitating systemic chemotherapy as the preferred treatment modality.^[12]^ Most experts no longer advocate radiotherapy due to the potential risk of long-term sequelae.^[9]^ Recent suggestions that LCH may be a neoplastic disorder have prompted reconsideration of patients with severe LCH as candidates for innovative targeted therapies. Promising therapeutic options include RAF, MEK1 inhibitors, BRAF inhibitors, and clofarabine.^[3,9,13]^

Given the nature of the lesion, some scholars recommend that a simple curettage during the diagnostic biopsy can lead to complete healing, eliminating the need for further intervention.^[9]^ The lesions did not encroach upon the orbit, and no other lesions were detected during the comprehensive examination. We successfully excised the lesions and utilized a micro-dynamic system to remove the affected bone. The impact of systemic therapy on children remains inadequately documented. Consequently, we advocate for a wait-and-see approach, with regular checkups and the avoidance of additional interventions. Due to differences of opinion on the treatment, pediatricians consider this invasion as a high-risk factor for this disease. However, in our opinion, administering systemic therapy is not advisable due to its associated side effects. LCH at the infraorbital margin differs from LCH at the superior or superolateral orbital roof since it is superficial, observable, and poses no risk of brain spread. In case of local recurrence, re-surgery causes less trauma and minimal risk to the eyes. Therefore, We advocate the systemic therapy should be reserved only if necessary.

Prognostic factors for LCH are well-established and encompass the extent of the disease at the time of diagnosis, the presence of organ dysfunction, and early response to therapy.^[3]^ While patients without organ dysfunction typically exhibit excellent survival rates, patients with organ dysfunction may face mortality rates ranging from 30% to 40%. Even for patients with low-risk disease, where a cure is almost assured, disease reactivation rates exceed 30%.^[10,14]^ Hence, it is imperative to closely monitor all cases to detect local recurrences and identify potential systemic lesions promptly.

In conclusion, LCH rarely occurs at the infraorbital margin. Hematomas developing at this location may be associated with LCH, making local bleeding more likely following exposure to external factors. These factors, including trauma, can induce localized bleeding and the formation of hematomas, potentially concealing the presence of underlying lesions and leading to missed diagnoses. Thus, it is essential to identify radiographic features, such as localized worm-eaten bone changes, to facilitate the early detection of LCH and to offer the optimal treatment strategy.

## Author contributions

**Conceptualization:** Jing Li, Mingyu Ren.

**Data curation:** Jing Li, Mingyu Ren.

**Formal analysis:** Mingyu Ren, Yanyan Cheng.

**Investigation:** Jianjie Wang, Ruimiao Li, Lu Lu.

**Methodology:** Jing Li, Ruina Zhang.

**Project administration:** Mingyu Ren.

**Supervision:** Jing Li, Mingyu Ren.

**Writing – original draft:** Jing Li, Yanyan Cheng.

**Writing – review & editing:** Mingyu Ren.
